# Cytogenetic investigation of Arctic char × brook trout F_1_, F_2_ and backcross hybrids revealed remnants of the chromosomal rearrangements

**DOI:** 10.1007/s13353-020-00584-2

**Published:** 2020-10-31

**Authors:** Konrad Pomianowski, Konrad Ocalewicz

**Affiliations:** 1 Department of Genetics and Marine Biotechnology, Institute of Oceanology, Polish Academy of Sciences, ul. Powstańców Warszawy 55, 81-712 Sopot, Poland; 2 Department of Marine Biology and Ecology, Faculty of Oceanography and Geography, Institute of Oceanography University of Gdansk, al. Marszalka Józefa Pilsudskiego 46, 81-378 Gdynia, Poland

**Keywords:** Chromosome fragments, Fish, Internal telomeric sites (ITS), Interspecific hybrids, *Salvelinus*, Triploid fish

## Abstract

Arctic char (*Salvelinus alpinus*) and brook trout (*Salvelinus fontinalis*) hybridize and their offspring is viable and fertile. This may be a real treat for the native European stocks of Arctic char which gene pools might be unintendedly contaminated with the genetic elements of brook trout. On the other hand, hybrids of these two species are appreciated by customers and have some potential for the aquaculture. Moreover, *Salvelinus* hybrids and backcross individuals are interesting models in the research focused on influence of hybridization on the genomic organization and chromosome rearrangements. Thus, the main goal of the present study was to examine chromosomes of Arctic char × brook trout F_1_, F_2_ hybrids and backcross individuals and compare with genomic information concerning parental species to recognize karyotypic changes provoked by the hybridization events. Application of conventional and molecular (FISH) techniques allow to identify characteristic chromosomes for both parental species in the hybrid progeny and show multiplicity of cytotypes among different types of crosses with variability in structure and number of chromosome (81–85) and chromosome arm (99–101). Chromosome fragment was detected in the karyotype of one F_1_ and one backcross individual and the presence of one triploid (3n) fish was documented. Occurrence of chromosomes containing internally located telomeric sequences (ITS) inherited after brook trout or both parental species was shown in F_1_ and backcross progeny. Moreover, additional CMA_3_-positive signal on chromosome from Arctic char pair no. 2 in F_1_ fish and interstitially located active NOR visible on subtelo-acrocentric (F_2_ hybrid) and acrocentric (Sf × H individual) chromosomes were detected. Described polymorphic chromosomes together with specific, interstitial location of CMA_3_-positive found in F_2_ and Sf × H hybrids and DAPI-positive regions observed in H × Sa fish at different uniarmed chromosomes pair presumably are remnants of chromosomal rearrangements. Provided results strongly indicate that the hybridization process influenced the genome organization in the *Salvelinus* hybrid progeny.

## Introduction

Arctic char (*Salvelinus alpinus* Linnaeus, 1758) and brook trout (*Salvelinus fontinalis* Mitchill, 1714) are freshwater char species, members of the genus *Salvelinus*. Arctic char is the most northern distributed among chars (Johnson 1980) ranging from Canada and Greenland to northern Russia and Europe (Iceland and Scandinavia) (Kottelat and Freyhof 2007). Brook trout is indigenous to North America and Canada and has been widely introduced to South America, Europe, Asia and Southern Africa (Kottelat and Freyhof 2007). Both species may exhibit different morphs (benthic and pelagic) and ecological forms: freshwater landlocked populations (lacustrine and riverine) and anadromous stocks (Jonsson and Jonsson 2001; Klemetsen et al. 2003; Morinville and Rasmussen 2003).

Under natural conditions, Arctic char and brook trout easily hybridize and reciprocal crosses of these two species, F_2_ hybrids and backcrosses are viable and usually fertile (Hammar et al. 1991). As a consequence, introgressive hybridization leading in incorporation of Arctic char mitochondrial genome into brook trout was reported in the nature (Bernatchez et al. 1995; Glémet et al. 1998). Moreover, Faulks and Östman (2016) found the evidence for hybridization of native Arctic char and introduced brook trout populations where majority of fish were F_2_ hybrids with mtDNA of both species. The ease with which both *Salvelinus* species hybridize can be a real treat for the European stocks of Arctic char which gene pools might be contaminated with the genetic elements of brook trout that escaped from the aquaculture farms (Castillo et al. 2008; Perrier et al. 2013).

On the other hand, both Arctic char and brook trout represent an aquaculture importance (Fischer et al. 2009; Sæther et al. 2013) and *Salvelinus* crosses are considered to have potential for cultivation under the control conditions. Aquaculture sector, when looking for fish that show unique characteristics and traits, cross different species producing F_1_, F_2_ and backcross hybrids. Some of these hybrids are produced to meet particular expectations of the customers such as meat colour, structure, and taste or because they possess better performances of growth rate or survival when compared with the parental species, among others (Chevassus 1979; Kerr 2000; Suzuki and Fukuda 1972, 1973). *Salvelinus* fish including hybrids are more resistant for the viruses infections than rainbow trout (*Oncorhynchus mykiss*) what make them better for the aquaculture in regions with frequent outbreaks of VHS disease (Dorson et al. 1991). Moreover, hybrids of brook trout and Arctic char show better growth rate than parental species (Sutterlin et al. 1977; Suzuki and Fukuda 1972) as well as better total biomass production and lower mortality after 3 years post-hatch than brook trout (Dumas et al. 1996). In Germany, Arctic char × brook trout hybrids known as “Elsässer Saibling” are famous by its exceptional taste (Piwernetz 2002).

Such hybrids are also a valuable material for research concerning influence of hybridization on the genomic organization and chromosome rearrangements. In the present research, we investigated karyotypic and genomic changes in the F_1_ and F_2_ brook trout and Arctic char hybrids and in the backcross individuals produced under the aquaculture conditions. Both species are closely related; however, their karyotypes present some differences. A rather stable karyotype of brook trout (2n = 84, NF = 100) (Fujiwara et al. 1998; Hartley 1987; Ocalewicz et al. 2004) contrasts with chromosome and chromosome arm number polymorphism (2n = 74–84, NF = 98-100) observed in the Arctic char from different locations (Phillips and Ihssen 1985; Pomianowski et al. 2012; Reed and Phillips 1997; Vasiliev 1975). Moreover, both species show sometimes unique genomic location of GC- and AT-rich chromatin, nucleolar organizer regions (NORs) or telomeric DNA sequences (Ocalewicz et al. 2004; Pomianowski et al. 2012; Śliwińska-Jewsiewicka et al. 2015; Table 1). Thus, it has been tempting to study changes in the chromosome and chromosome arm numbers, chromosome structures, NORs, distribution of DAPI- and CMA_3_-positive regions, and telomeric DNAs in Arctic char and brook trout F_1_ and F_2_ hybrids and backcrosses.

**Table 1:** Comparative cytogenetic analysis of Arctic char *S. alpinus* and brook trout *S. fontinalis* from Inland Fisheries Institute broodstock

Species	2n	NF	Major rDNA AgNOR	Major rDNA 18/28S rDNA	Minor rDNA (5S)	Internal telomeric sites (ITS)	Heterochromatin C-band, GC-rich or AT-rich	Sex chromosomes	Reference
*Salvelinus alpinus*	81–82	100 Robertsonian polymorphism	1–6 sites			**Characteristic chromosome**: 10q (ITS)	Interindividual variation in number and size of GC- and AT-rich blocks **Characteristic chromosome: 2p, 6q and 8q**		Pomianowski et al. 2012
*Salvelinus fontinalis*	84	100	3–6 sites **Characteristic chromosome: 29 p;q**	Multichromosomal (24)	Pair no. 9 not associated with NOR	**Characteristic chromosome**: 11p (ITS)	GC-rich: polymorphic in size and number at 10–20 sites per cell **Characteristic chromosome 3q and 9p**	XY pair no. 5 DAPI-positive	Śliwińska-Jewsiewicka et al. 2015
*Salvelinus fontinalis*	84	100			1 pair not associated with NOR GC/DAPI-positive	**Pair of characteristic chromosomes**: 11p (ITS)		XY pair no. 5 DAPI-positive	Ocalewicz et al. 2004

## Materials and methods

### Material

Char (*Salvelinus*) hybrid lines (Table 2) were obtained as a result of crossing of brook trout and Arctic char: F_1_ (*S. alpinus* × *S. fontinalis*), F_2_ (*S. fontinalis* × *S. alpinus*) × (*S. fontinalis* × *S. alpinus*), backcross *S. fontinalis* × (*S. fontinalis* × *S. alpinus*) and reverse cross; *S. alpinus* × (*S. fontinalis* × *S. alpinus*). All fish were bred and kept at the Department of Salmonid Research, Inland Fisheries Institute in Olsztyn, Rutki, Poland. In total, 92 fish were sampled from 0+,1+,2+ individuals at 2000–2020.

**Table 2:**
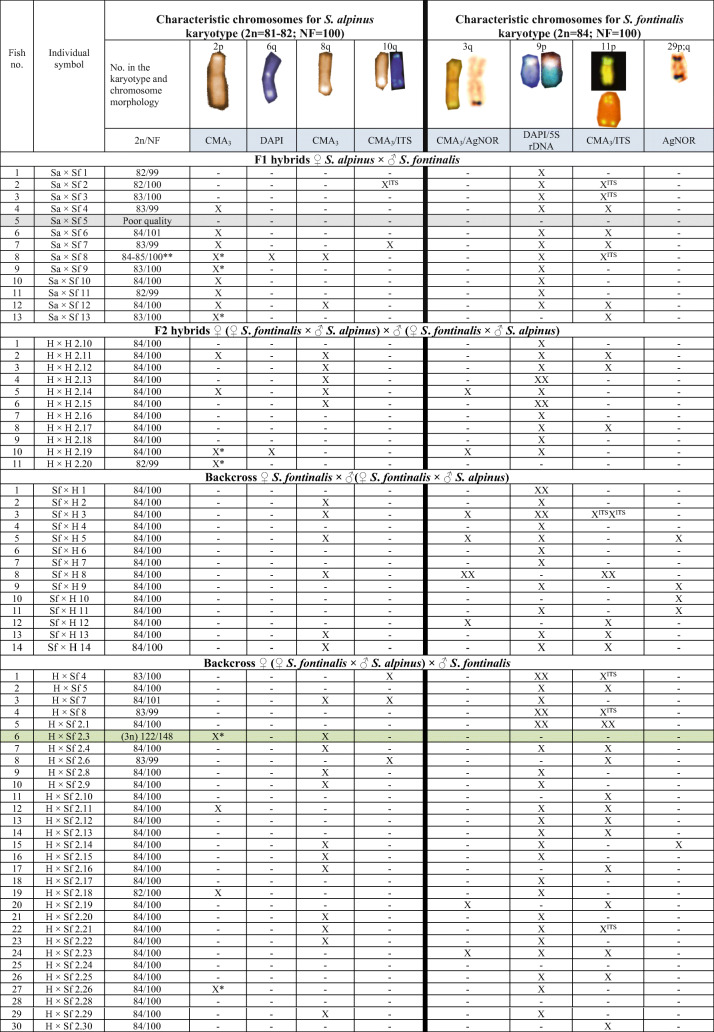

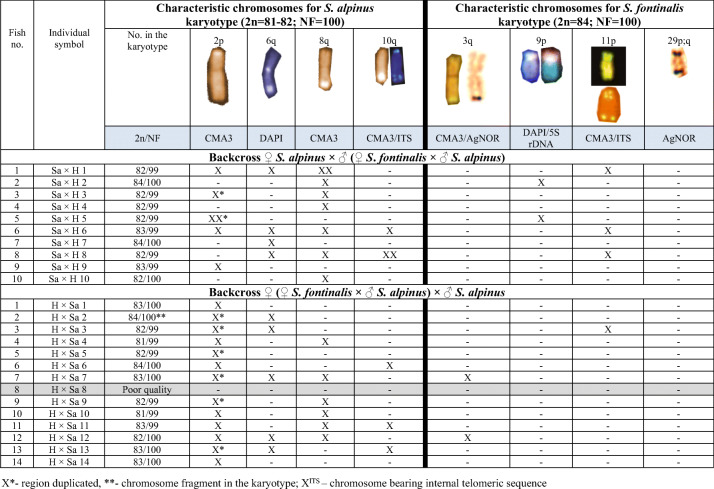
Diploid chromosome number (2n), chromosome arm number and chromosomes characteristic of the parent brook trout and Arctic char species from Inland Fisheries Institute broodstock occurring in the F1, F2 and backcross generations

### Methods

#### Chromosome preparation

Fish were sacrificed and metaphase spreads were obtained from the head kidney tissues using conventional in vivo “splash” technique according to Ráb and Roth (1988). Total number of 2510 metaphase plates were analysed among all studied fish.

#### Banding techniques, FISH protocols and image processing

Chromosomes were initially stained with the buffered Giemsa solution (10%, 10 min) for visualization and description of the chromosome morphology. Active nucleolus organizer regions (NORs) were visualized using impregnation with silver nitrate (AgNO_3_) as described by Howell and Black (1980). Chromomycin A_3_ (CMA_3_) fluorochrome staining was used to identify GC-rich chromosomal regions (Sola et al. 1992). For identification of AT-rich chromatin bearing sites, chromosomes were stained with 4,6-diamidino-2-phenylindole (DAPI) using antifade solution Vectashield with 1.5 μg/ml DAPI (Vector, Burlingame, CA). Fluorescence in situ hybridization (FISH) technique was applied for detection of telomeric DNA repeats using a telomere PNA (peptide nucleic acid) probe conjugated with FITC (DAKO, Denmark) according to the manufacturer’s protocol with some modifications (Ocalewicz et al. 2013a). Briefly, chromosomal DNA was denaturated at 85 °C for 5 min under the coverslip in the presence of the telomere PNA probe. Hybridization took place in the darkness at room temperature for at least 40 min. Chromosomes were counterstained with 25 μl Vectashield with DAPI. Metaphase plates were examined under a Zeiss Axio Imager.A1 microscope equipped with a fluorescent lamp and a digital camera. Images were captured and the electronic processing of the images was performed using Band View/FISH View software (Applied Spectral Imaging).

## Results

### Size and position polymorphism of NORs, GC- and AT-rich chromatin

#### F_1_ hybrids *♀ S. alpinus × ♂ S. fontinalis*

**Fig. 1:**
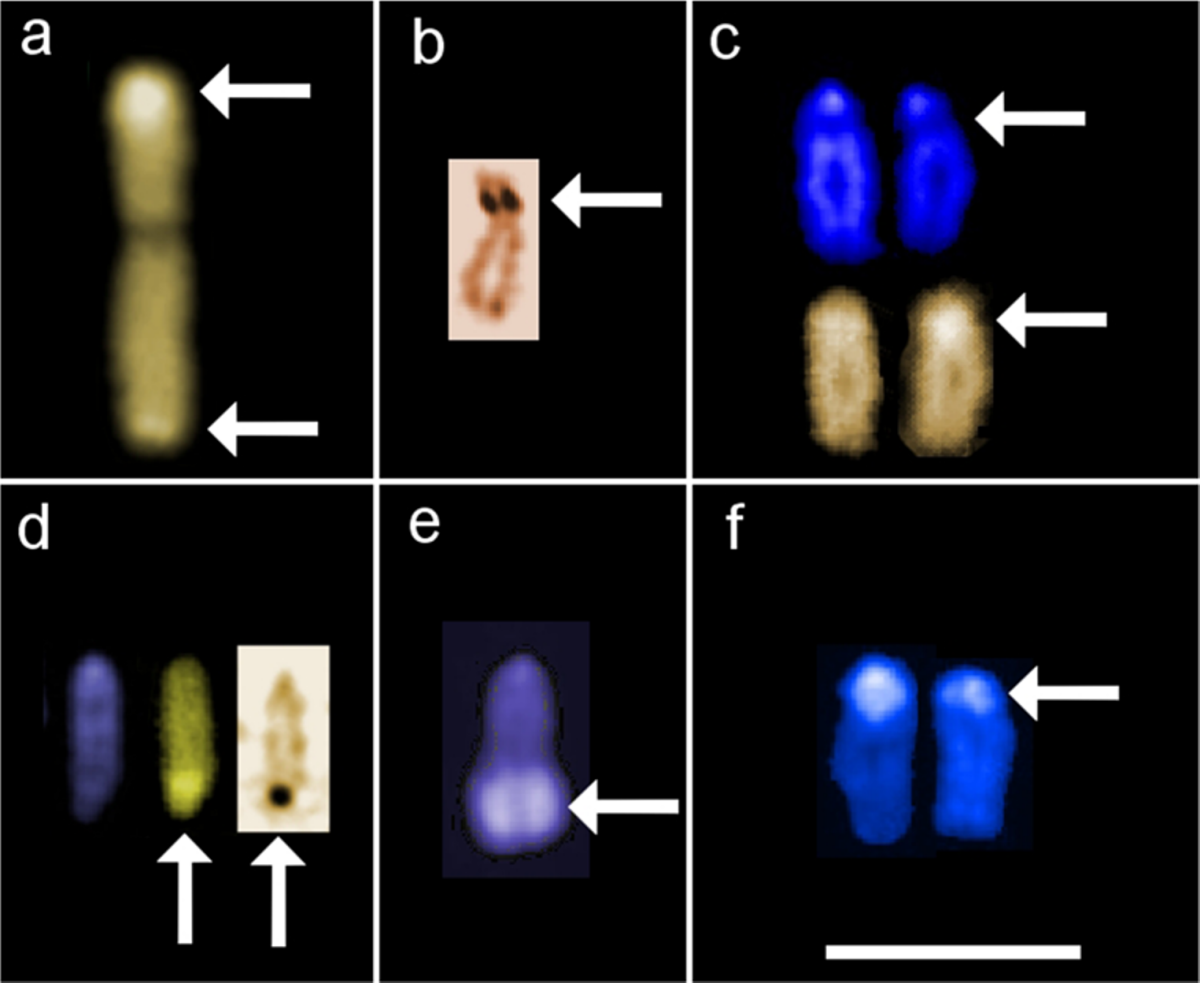
Polymorphic location of AgNOR, GC- and AT-rich regions on investigated fish chromosomes: **a** F_1_ Sa × Sf fish: chromomycin A_3_ staining revealed chromosome from Arctic char pair no. 2 with additional CMA_3_-positive signal on the long arm; **b** F_2_ fish: subtelo-acrocentric chromosome with interstitially located active NOR region (p arm); **c** F_2_, backcross Sf × H fish: upper row—DAPI staining, lower row—chromomycin A_3_. Arrows indicate GC-rich chromatin located interstitially at the proximal part of the long arm; **d** backcross Sf × H fish: sequentially stained DAPI (left), chromomycin A_3_ (in the middle) and silver nitrate (right) acrocentric chromosome with interstitially located active NOR region colocated with CMA_3_-positive region in the distal part of the long arm; **e** Backcross H × Sf fish: arrows indicate large acrocentric chromosome with duplicated AT-rich region revealed by DAPI staining **f** backcross H × Sa fish: DAPI staining revealed AT-rich region located interstitially at the proximal part of the long arm (marked by arrows) of uniarmed chromosomes pair. Bar equals 5 μm

AgNO_3_ staining revealed from one to five positive signals on metaphase spreads located in the telomeric area of the large a chromosome or on medium-sized st-a chromosomes on the p arm, where duplication of the region was visible in one fish. CMA_3_ staining showed from two to seven positive signals in the examined fish. Signals were located in the end of both p and q arms of 2p chromosome (Fig. 1a) as well as on large- and medium-sized a chromosomes in the end of q arm. In examined individuals from two to seven chromosomes with DAPI, positive sites (AT-rich) have been observed. Described regions were placed on m chromosomes in the end of p and q arm (one fish) or only in telomeric area of q arm, and on st-a chromosome (p arm) or a chromosomes (end of q arm). DAPI staining revealed one chromosome fragment in the karyotype of one individual (Table 2) (Fig. 5a).

#### F_2_ hybrids *♀* (*♀ S. fontinalis × ♂ S. alpinus*) *× ♂* (*♀ S. fontinalis × ♂ S. alpinus*)

In F_2_, individuals from three to seven AgNOR sites were seen on chromosomes. They were located in the end of the long arm of medium-sized m and a chromosomes as well as on st-a chromosomes on p arm where duplication of the region (one fish) and its interstitial location (other individual) was observed (Fig. 1b). CMA_3_ signals were seen on from three to six chromosomes. GC-rich chromatin was located on st-a chromosomes in the end of p or both p and q arms and on acrocentric chromosomes where CMA_3_-positive site was seen in the end of the long arm. Pair of uniarmed chromosomes with interstitially, subcentromeric location of signal was seen in one fish (Fig. 1c). From one to three DAPI, positive signals were detected in that type of cross located on p and q or only on long arm of the m chromosomes (Fig. 5b).

#### Backcross *♀ S. fontinalis × ♂* (*♀ S. fontinalis × ♂ S. alpinus*)

AgNO_3_ staining revealed from one to eight positive signals on metaphase spreads. Active NORs were located on st-a chromosomes in the end of short arm where overlapped with CMA_3_ signal in two different individuals, and on uniarmed chromosomes in telomeric area of the long arm (q). Moreover, active NORs were detected on interstitial, subtelomeric location on large a chromosome in one individual where overlapped with CMA_3_ signal (Fig. 1d). CMA_3_ staining revealed from three to six CMA_3_-positive signals in the examined individuals. GC-rich sites were detected in the end of q arm of m and a chromosomes and on st-a chromosomes in telomeric area of p arm. Moreover, as in F_2_ generation pair of uniarmed chromosomes with interstitially, subcentromeric location of CMA_3_-positive signal was seen in one fish (Fig. 1c). There were from one to three DAPI signals visible on chromosomes revealing AT-rich chromatin on medium-sized m chromosome in the end of q arm apart from single or pair 9p (Fig. 5c).

#### Backcross *♀* (*♀ S. fontinalis × ♂ S. alpinus*) *× ♂ S. fontinalis*

**Fig. 2:**
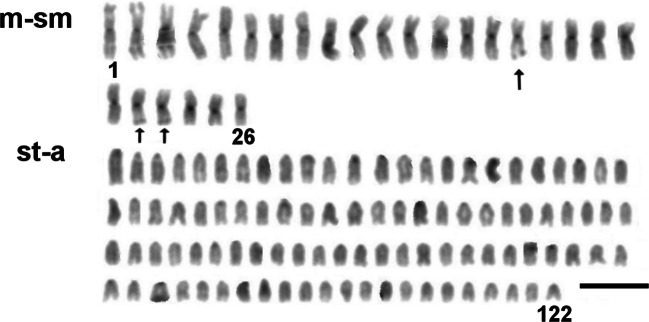
Karyotype of the backcross H × Sf individual with total chromosome number 3n = 122. Chromosomes stained with DAPI fluorochrome. Arrows marked chromosomes containing regions rich in AT base pairs. Bar equals 10 μm

In described backcross, AgNO_3_ staining revealed from one to six signals on metaphase spreads. Active NOR sites were visible on large m chromosome in the end of long arm (q) and on varying in size st-a chromosomes in the telomeric region of p arm where duplication was visible in one fish. Moreover, AgNOR sites were visible in the end of the long arm of a chromosomes where duplication was detected in one fish. From two to four, CMA_3_ signals were seen in that type of cross. GC-rich sites were located on varied in size: st-a chromosomes in the end of long arm and in the telomeric area of the uniarmed chromosomes. In one individual, interstitial, subcentromeric location of CMA_3_ signal was detected on single a chromosome, and its morphology corresponded with chromosome pair detected in F_2_ generation. DAPI staining revealed from two to three AT-rich sites on chromosomes, in the majority of individuals located at the end of the long arm of two (diploids) or three (triploids) medium-sized m chromosomes (Fig. 2). In four fish, large uniarmed chromosome with DAPI-positive signal reaching half of the q arm was visible (Fig. 1e, Fig. 5d).

#### Backcross ♀ *S. alpinus* × ♂ (♀ *S. fontinalis* × ♂ *S. alpinus*)

In described individuals from two to six, active NOR sites were presented. NORs were placed on pair of large, medium-sized or single st-a chromosomes in the end of p arm and on single large and pair of small a chromosomes in the telomeric area of the long (q) arm. CMA_3_ staining showed from two to eight GC-rich sites. Regions were visible on m chromosome in two fish placed in the end of p and q arm, and on the large, medium-sized, and small st-a chromosomes in the end of long arm. Additionally, described sites were located on large and small a chromosomes in the end of the long arm. DAPI staining showed from one to two AT-rich signals on metaphase spreads located on large m and a chromosomes in the end of q arm (Fig. 5e).

#### Backcross ♀ (♀ *S. fontinalis* × ♂ *S. alpinus*) × ♂ *S. alpinus*

**Fig. 3:**
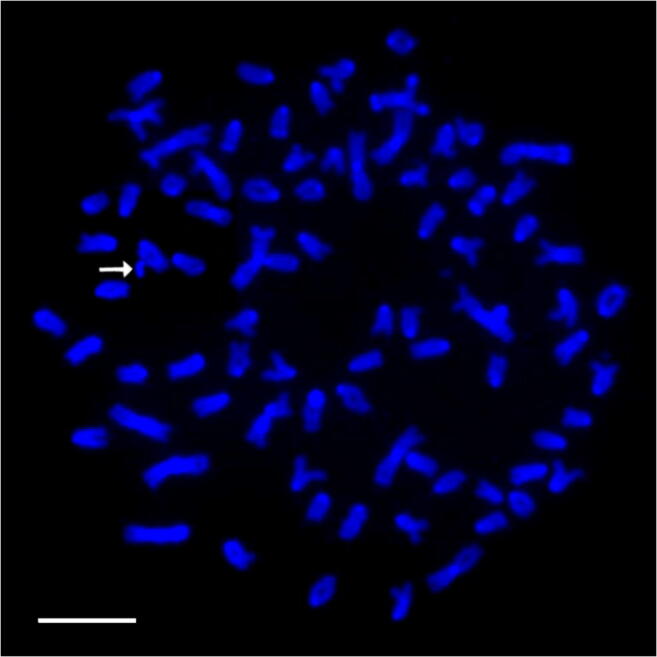
Metaphase plate of backcross H × Sa after DAPI staining. Arrow indicates chromosome fragment. Bar equals 10 μm

In described cross number of active NOR ranged from one to four. NORs were placed on large m chromosome in the end of the long (q) arm and on large, medium-sized and small st-a chromosomes in the end of p arm where duplication of the region was detected in one fish. Additionally, AgNORs were visible on large and small a chromosomes in the end of the long arm in most of the karyotyped fish. CMA_3_ staining revealed from two to five signals in the described metaphase spreads. Apart from GC-rich regions located on recognizable brook trout and Arctic char chromosomes, described sites were placed on large st-a chromosome in the end of long arm and on large and small a chromosome (end of q arm). DAPI staining showed from one to three signals on investigated metaphase spreads. AT-rich sites were located in the end of q or both arms of m-sm chromosome or in the end of q arm on a chromosome. In few individuals, pair of large acrocentric chromosomes with interstitially, subcentromerically located DAPI-positive blocks was evidenced (Fig. 1f). DAPI staining revealed one chromosome fragment in the karyotype of one individual (Fig. 3, Fig. 5f).

#### Fluorescence *in situ* hybridization

**Fig. 4:**
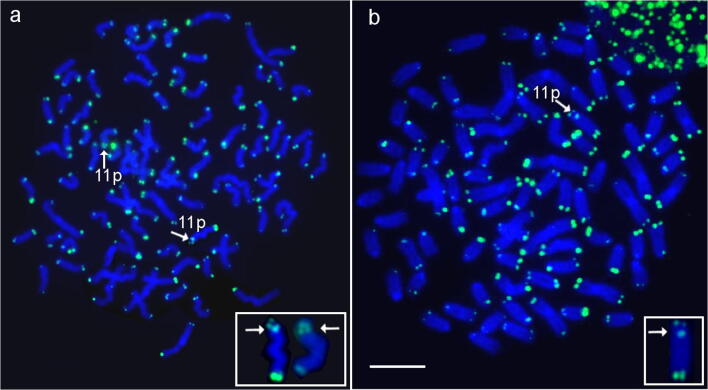
Metaphase spreads after application of PNA-FISH with telomeric probe. Arrows point on **a** pair (backcross Sf × H) and **b** single (backcross H × Sf) brook trout chromosomes 11p containing internally located telomeric sequence (ITS). Bar equals 10 μm

Application of PNA-FISH with telomeric probe in the reciprocal *S. fontinalis* × H backcross individuals showed hybridization signals at the terminal positions of all chromosomes among studied fish. Moreover, one member of *S. fontinalis* × H cross had two chromosomes with internally located telomeric signals (ITSs) (Fig. 4a). ITSs were located interstitially on the p arm of the pair of st chromosomes that was *Salvelinus fontinalis* chromosomes no. 11 (Śliwińska-Jewsiewicka et al. 2015). Similarly, ITS signal was detected on single chromosome of the same type in three members H × *S. fontinalis* cross (Fig. 4b).

## Discussion

Hybrids of brook trout and Arctic char are appreciated by the customers; however, the ease in which F_1_ individuals are able to mate and to create post-F_1_ generations may negatively affect valuable broodstocks of parental species when unintendedly backcrossed. Indeed, F_1_ and post-F_1_ hybrid swarm (83–100%) was confirmed in several hatchery stocks (Gross et al. 2004). On the other hand, F_1_ char hybrids may be used for the reconstitution of a genetic strain of the paternal species by programme of repeated backcrossing. Such method might be considered to recover genetic information from the fish cryopreserved spermatozoa from the extinct population or even species (Ocalewicz et al. 2013b). Although recovery of the gene pool from the repeated backcrossing takes a relatively long time, it is still a more efficient approach than an interspecies androgenesis process (Babiak et al. 2002; Ocalewicz et al. 2013b; Michalik et al. 2014). Interspecies androgenesis fails because of interspecies incompatibilities between egg cytoplasm and sperm genome. Application of eggs originated from Arctic char and brook trout hybrids seemed to overcome this problem and androgenetic development of brook trout in such oocytes was possible (Ocalewicz et al. 2013b). Taking into account the research and results described above, it seems to be indispensable to provide cytogenetic investigation among Arctic char × brook trout F_1_, F_2_ fish and backcross generations to assess the impact of hybridization on their genomic organization.

**Fig. 5:**
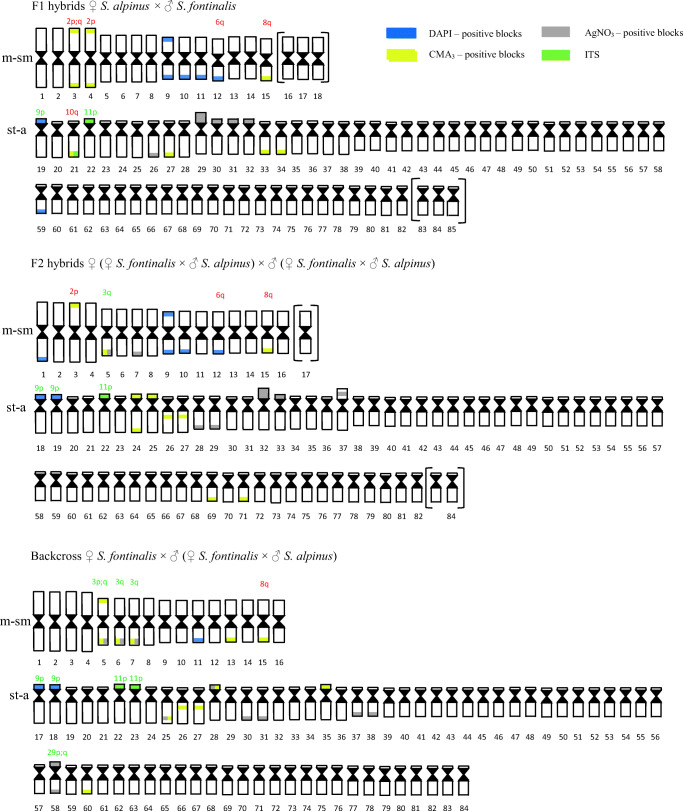

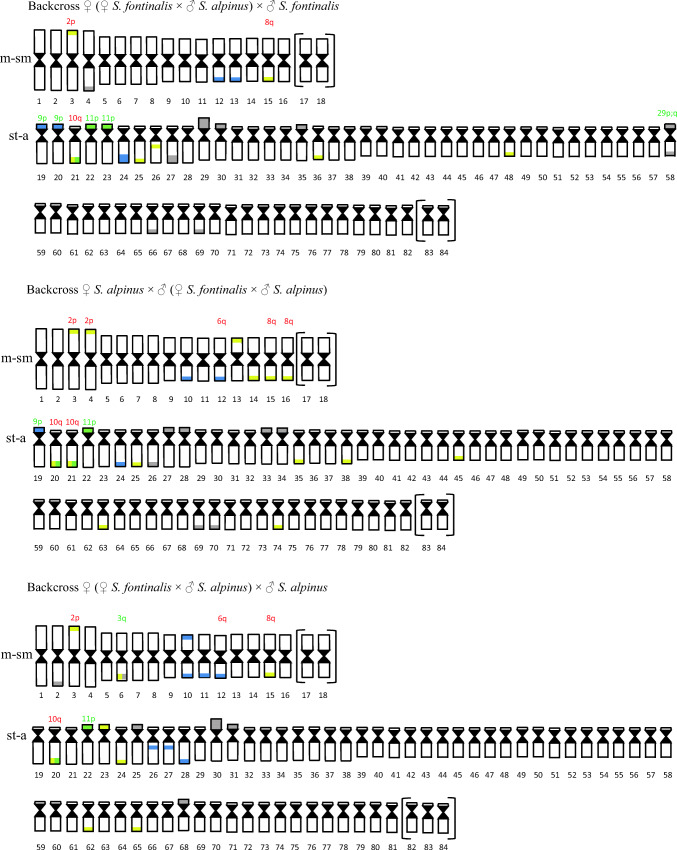
Ideograms of chromosomes from examined Arctic char and brook trout hybrids and backcrosses. Chromosomes characteristic for Arctic char and brook trout karyotypes are indicated by red and green symbols respectively pasted above particular chromosomes and corresponded with data collected in Table 2. Chromosomes in brackets are these that number varied in individuals with different chromosome numbers

Our data showing high variability in the karyotype structure among F_1_ individuals (2n = 81–85; NF = 99–102) (Fig. 5) contrasts with early reports presenting stable number of chromosomes (82) in F_1_ brook trout × Arctic char individuals (Disney and Wright Jr 1987). Arctic chars can be carriers of centric fusion(s) (Robertsonian polymorphism) (Pomianowski et al. 2012; Vasiliev 1975) what results in production of gametes containing varied numbers of chromosome. Occurrence of centric fusion in one of the parental species as well as de novo centric fusion and fission (decreasing/increasing chromosome numbers) may be responsible for the multiplicity of cytotypes in F_1_ and backcross generations. In contrast, one stable karyotype variant was detected in most of the F_2_ individuals what indicates that some variants are supported.

In the H × Sf backcross group, one individual appeared to be triploid and possessed 122 chromosomes (26 m and 96 a) and might appear in the course of fertilization of an unreduced F_1_ hybrid egg containing 18 bi- and 66 uniarmed chromosomes by brook trout male gamete (8 bi- and 34 uniarmed chromosomes) followed by elimination of four chromosomes. Triploids were previously described in several crosses of fish (Boroń 2003; Hashimoto et al. 2014) including F_1_ *S. fontinalis* × *S. alpinus* hybrids that karyotype was composed of 124 chromosomes: 26 metacentrics and 98 acrocentrics (Disney and Wright 1987). The cases of spontaneous triploidy were previously described in rainbow trout (Aegerter and Jalabert 2004; Ocalewicz and Dobosz 2009; Thorgaard et al. 1982) and Atlantic salmon (Glover et al. 2015) under hatchery conditions. The post-ovulatory ageing of eggs and their reduced quality are considered to be responsible for spontaneous triploidization (Aegerter and Jalabert 2004).

Both parental species of hybrids show interspecific differences in the location of NORs, GC- and AT-rich blocks of chromatin (Fujiwara et al. 1998; Phillips and Ráb 2001). Number of active NORs in the genome of Arctic char varies from one to eight depending on the location, whereas brook trout represents from three to six AgNO_3_-positive sites (Phillips and Ihssen 1985; Reed and Phillips 1997; Śliwińska-Jewsiewicka et al. 2015). In Arctic char, NORs are typically distributed on p arm of one m-sm pair and on m-sm and a chromosomes (Phillips et al. 1988) whereas in brook trout NORs are observed on single m chromosome and pair and single st-a chromosomes (Fujiwara et al. 1998; Śliwińska-Jewsiewicka et al. 2015). AgNO_3_ sites do not always overlap with CMA_3_-positive sites, which number in Arctic char equals from two to eight (Phillips et al. 1988; Pomianowski et al. 2012) and in brook trout varied from 10 to 20 sites (Phillips and Ihssen 1985; Śliwińska-Jewsiewicka et al. 2015). Variation in the number and location of NORs in parental species leads to NOR polymorphism in the progeny. In F_1_ fish: Sa × Sf and reciprocal cross number of active NORs varied from one to eight, whereas Disney and Wright Jr (1987) detected NOR sites on up to five chromosomes (m, a) in Sf × Sa fish what may result from different origin of parents of hybrids. Moreover, authors did not report NOR size variation as in the fish described here. Visible bi-armed chromosomes with NOR located at the ends of both arms (pair no. 2 in Sa × Sf fish) are remnants of rearrangements in the progeny. The mechanism leading to described location was proposed by Reed and Phillips (1995) in *Salvelinus namaycush* where st-a chromosome with multiple NOR was detected in the karyotype. Described chromosome may be a result of translocation of chromosomal fragment carrying rRNA genes from telomeric region of the uniarmed chromosome to proximal part of its homologue. In a similar way, chromosome 2p;q could have been formed if the translocation had occurred between the metacentric chromosome containing NOR at the end of p arm and the uniarmed chromosome with NOR placed in the same area. In part of fish from F_2_ and backcross generation, interstitial location of NOR was shown on st-a chromosome. Such specific location might have been a result of pericentromeric inversion in the *S. alpinus* large bi-armed chromosome with partial deletion of the short arm of newly created chromosome. In one Sf × H individual, large a chromosome with interstitially, subtelomerically located CMA_3_-positive signal overlapped with active NOR. Similar chromosome was described in Arctic chars from aquaculture broodstock (Pomianowski et al. 2019). It may have appeared in the course of pericentromeric inversion of st-a chromosome with NOR located on the short arm. Finally, we described for the first time in *Salmonidae* family single (H × Sf) and pair (H × H, Sf × H) of large uniarmed chromosomes with unusual, interstitial subcentromeric location of CMA_3_-positive signal. Its origin is probably the result of tandem fusion of two a chromosomes with loss of one centromere.

In members of the genus *Salvelinus*, DAPI-positive heterochromatin blocks are usually visible on centromeric and telomeric regions of many chromosomes (Hartley 1989; Mayr et al. 1988; Pleyte et al. 1989; Ueda and Ojima 1983). Described location was revealed in the majority of studied fish except H × Sa individuals, where pair of large acrocentrics with interstitially located DAPI band is observed and in H × Sf individuals possessing large uniarmed chromosome with DAPI signal reaching half of the arm. Both chromosomes were previously reported in the farmed Arctic chars (Pomianowski et al. 2019) and taking into consideration their size, these chromosomes appeared in the course of the tandem fusion. Tandem fusions are rearrangements that affect number of chromosomes and chromosome arms and are responsible for differences in the chromosome number in European (2n = 54) and North American (2n = 58) populations of Atlantic salmon (*Salmo salar*) (Hartley and Horne 1984a, 1984b; Ueda and Kobayashi 1990). In the karyotypes of Atlantic salmon from European population, interstitial DAPI signals were located on pair of bi-armed chromosomes and, similarly as in investigated hybrids, on the long arms of pair of a chromosomes (Phillips 2005). Tandem fusions are also responsible for karyotype variability in *Oreochromis niloticus* and *O. karongae* (Chew et al. 2002; Mota-Velasco et al. 2010).

Internally located telomeric DNA sequences usually appeared as a result of chromosome rearrangements including fusions and inversions (Mota-Velasco et al. 2010; Ocalewicz et al. 2013a; Perez et al. 1999). In *Salmonidae*, ITSs have been detected on chromosome(s) of rainbow trout (*Oncorhynchus mykiss*) and Atlantic salmon (*Salmo salar*) (Abuin et al. 1996), lake trout (*Salvelinus namaycush*) (Reed and Phillips 1995), brook trout (Ocalewicz et al. 2004; Śliwińska-Jewsiewicka et al. 2015), Arctic char (Pomianowski et al. 2012) and European grayling (Ocalewicz et al. 2013a). In the brook trout, ITS are observed on p-arm of one or two subtelomeric chromosomes, adjacent to the GC-rich nucleolar organizer region (Ocalewicz et al. 2004; Śliwińska-Jewsiewicka et al. 2015), whereas in Arctic char from Rutki strain ITS was detected at the subtelomeric region of the q arm of the largest acrocentric chromosome (Pomianowski et al. 2012). Here, in F_1_ generation hybrids (Sa × Sf), we confirmed the presence of single ITS bearing chromosomes inherited after both *Salvelinus* parental species. Presence of the same ITS bearing chromosomes was previously described in one Arctic char individual from the aquaculture broodstock (Pomianowski et al. 2019) what indicates past hybridization events during breeding practices. Identification of two brook trout chromosomes with ITS in Sf × H backcross, and one such chromosome in H × Sf fish, points on different type of chromosome selection during meiosis.

Genomic incompatibilities between parental species may result in increased mortality of the hybrid progeny. In Arctic char × brook trout F_1_ hybrids and Sa × H backcross individuals, survival rates before hatching reached 70.4% and 57.5%, respectively (Michalik et al. 2014; Ocalewicz et al. 2013b). In turn, F_2_ hybrids and backcrosses with genomic predominance of brook trout exhibited more than 90% of survival. In our study, presence of one chromosomal fragment in the karyotypes of Sa × Sf and backcross H × Sa fish was confirmed. It evidenced that nucleocytoplasmic incompatibility between eggs and sperm originated from different species may result in increased mortality of the hybrid progeny resulted from the chromosome elimination (Fujiwara et al. 1997; Sakai et al. 2007). Chromosome fragments were also visible in metaphase spreads obtained from inviable hybrids of rainbow trout and sea trout (Polonis et al. 2018). Moreover, chromosome fragments were also observed in Arctic chars from the aquaculture broodstock (Pomianowski et al. 2019).

Provided cytogenetic data confirmed usefulness of interspecific *Salvelinus* F_1_, F_2_ and backcross hybrids as an object on karyotype rearrangements research and their possible future use in fish stock restoration studies.
